# Supplementary material for: Green Extraction of Forsythoside A, Phillyrin and Phillygenol from Forsythia suspensa Leaves Using a β-Cyclodextrin-Assisted Method
Source: Molecules. 2022 Oct 19;27(20):7055. doi: 10.3390/molecules27207055 (PMC9610952; doi:10.3390/molecules27207055)
Supplement: Supplementary file 1 [file molecules-27-07055-s001.zip › molecules-1949773-supplementary.pdf]

Supplementary Material

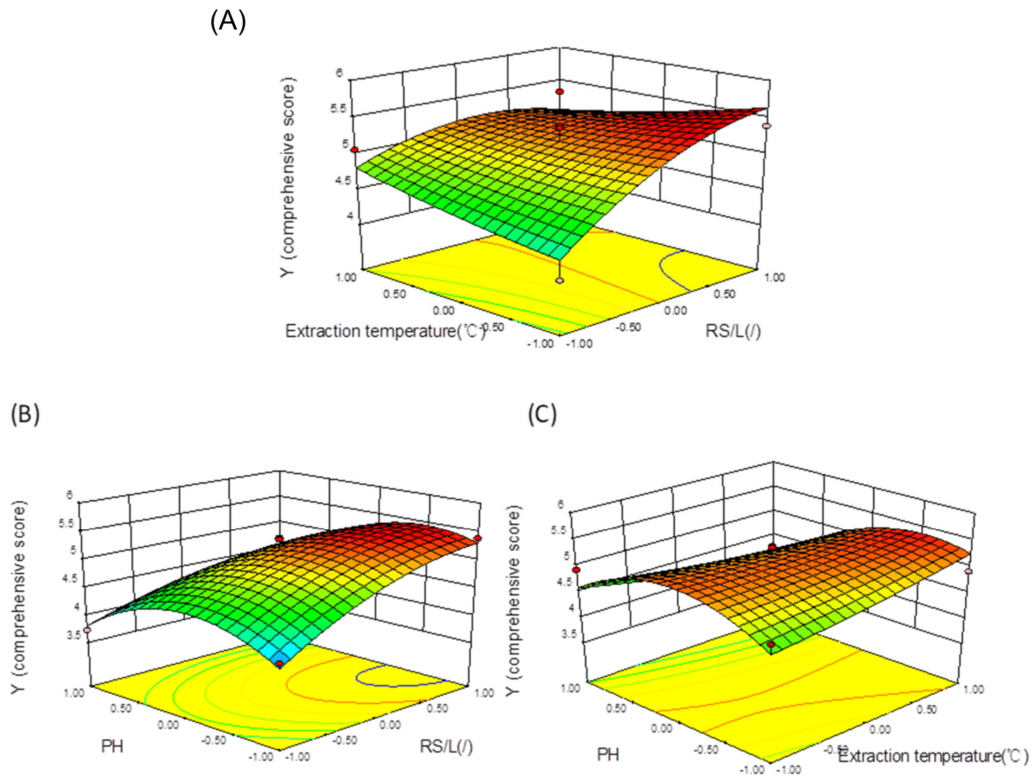

Figure S1. Three-dimensional response surface graphs of the influences of variables on Y(comprehensive score): (**A**) interaction of Extraction temperature and $R_{S/L}$; (**B**) interaction of pH and $R_{S/L}$; (**C**) interaction of pH and Extraction temperature.

Table S1. BBD and the response values of Y (comprehensive score) from *Forsythia suspensa* leaves.

Run	Independent variables				Extraction yield（%）			Y
	X_1 $R_{S/L}$(/)	X_2 Extraction temperature(℃)	X_3 pH	X_4 $R_{\beta/F}$ (g/g)	Forsythin A	Phillyrin	Phillygenol	
1	1:40(1)	55(-1)	3(0)	4:5(0)	11.89	5.48	0.31	6.49
2	1:40(1)	70(0)	3(0)	3:5(-1)	11.92	5.40	0.31	6.48
3	1:40(1)	70(0)	1(-1)	4:5(0)	10.60	4.91	0.35	5.82
4	1:30(0)	70(0)	5(1)	3:5(-1)	10.89	5.30	0.31	6.04
5	1:30(0)	85(1)	3(0)	5:5(1)	11.38	5.23	0.33	6.22
6	1:20(-1)	85(1)	3(0)	4:5(0)	9.55	4.89	0.32	5.38
7	1:20(-1)	70(0)	3(0)	5:5(1)	8.80	4.97	0.33	5.11
8	1:20(-1)	70(0)	3(0)	3:5(-1)	9.31	4.91	0.30	5.29
9*	1:30(0)	70(0)	3(0)	4:5(0)	11.81	5.40	0.32	6.44

10*	1:30(0)	70(0)	3(0)	4:5(0)	11.57	5.25	0.31	6.30
11	1:30(0)	85(1)	3(0)	3:5(-1)	11.71	5.36	0.32	6.39
12	1:20(-1)	70(0)	1(-1)	4:5(0)	8.62	3.95	0.31	4.73
13	1:30(0)	85(1)	1(-1)	4:5(0)	8.47	2.72	1.38	4.62
14	1:30(0)	85(1)	5(1)	4:5(0)	11.36	5.56	0.33	6.31
15	1:30(0)	55(-1)	3(0)	3:5(-1)	11.61	5.37	0.31	6.35
16*	1:30(0)	70(0)	3(0)	4:5(0)	11.52	5.28	0.31	6.29
17	1:30(0)	70(0)	1(-1)	5:5(1)	9.81	4.35	0.70	5.44
18	1:20(-1)	55(-1)	3(0)	4:5(0)	11.73	4.83	0.31	6.23
19	1:40(1)	70(0)	5(1)	4:5(0)	10.96	5.44	0.30	6.10
20	1:30(0)	55(-1)	1(-1)	4:5(0)	11.69	5.39	0.34	6.40
21	1:30(0)	70(0)	1(-1)	3:5(-1)	10.29	4.77	0.46	5.69
22	1:40(1)	85(1)	3(0)	4:5(0)	12.15	5.60	0.33	6.64

23*	1:30(0)	70(0)	3(0)	4:5(0)	11.86	5.51	0.32	6.50
24	1:20(-1)	70(0)	5(1)	4:5(0)	9.08	5.25	0.33	5.30
25	1:30(0)	55(-1)	5(1)	4:5(0)	11.45	5.63	0.33	6.37
26*	1:30(0)	70(0)	3(0)	4:5(0)	12.08	5.51	0.32	6.58
27	1:30(0)	55(-1)	3(0)	5:5(1)	10.41	5.51	0.33	5.91
28	1:40(1)	70(0)	3(0)	5:5(1)	11.96	5.45	0.32	6.51
29	1:30(0)	70(0)	5(1)	5:5(1)	9.93	5.61	0.33	5.75

* Indicated 5 center points

Table S2. Analysis of variance (ANOVA) for the developed second order polynomial models.

Source	Sum of squares	df	Mean square	F-value	P-value	
Model	8.05	14	0.58	7.81	0.0002	**
X_1	3	1	3	40.71	< 0.0001	**
X_2	0.4	1	0.4	5.43	0.0353	*
X_3	0.86	1	0.86	11.62	0.0042	**
X_4	0.14	1	0.14	1.88	0.1921	
X_1X_2	0.25	1	0.25	3.37	0.0877	
X_1X_3	0.021	1	0.021	0.2^{9}	0.6017	
X_1X_4	0.011	1	0.011	0.15	0.7045	
X_2X_3	0.74	1	0.74	10.05	0.0068	**
X_2X_4	0.018	1	0.018	0.25	0.6264	
X_3X_4	3.25E-04	1	3.25E-04	4.41E-03	0.948	

X_1^2	0.78	1	0.78	10.61	0.0057	**
X_2^2	0.02	1	0.02	0.27	0.6091	
X_3^2	1.88	1	1.88	25.55	0.0002	**
X_4^2	0.29	1	0.29	3.95	0.0668	
Residual	1.03	14	0.074			
Lack of Fit	0.97	10	0.097	5.96	0.0501	
Pure Error	0.065	4	0.016			
Cor Total	9.08	28				

Note: * represents significant difference in results ($p < 0.05$), ** represents extremely significant difference in results ($p < 0.01$).

Table S3. BBD and the response values of Y (comprehensive score) from pure water extraction *Forsythia suspensa* leaf.

Run	Independent variables			Extraction yield（%）			Y
	X_1 $R_{S/L}$(/)	X_2 Extraction temperature(℃)	X_3 pH	Forsythin A	Phillyrin	Phillygenol	
1*	1:20(0)	70(0)	5(0)	11.01	3.25	0.18	5.39
2	1:30(1)	55(-1)	5(0)	10.78	3.45	0.13	5.38
3	1:30(1)	70(0)	3(-1)	10.90	3.29	0.11	5.384
4	1:10(-1)	55(-1)	5(0)	9.33	1.07	0.02	4.06
5	1:20(0)	85(1)	3(-1)	10.45	2.39	0.05	4.91
6*	1:20(0)	70(0)	5(0)	10.29	3.16	0.03	5.07
7	1:20(0)	55(-1)	7(1)	8.61	3.78	0.17	4.93
8	1:10(-1)	70(0)	7(1)	8.20	1.51	0.003	3.73
9*	1:20(0)	70(0)	5(0)	10.84	2.35	0.024	5.05

10	1:30(1)	70(0)	7(1)	7.58	3.18	0.09	4.01
11*	1:20(0)	70(0)	5(0)	10.85	2.19	0.02	5.00
12	1:10(-1)	70(0)	3(-1)	9.49	1.13	0.002	4.13
13*	1:20(0)	70(0)	5(0)	10.88	3.18	0.05	5.36
14	1:20(0)	85(1)	7(1)	7.00	3.13	0.06	3.76
15	1:30(1)	85(1)	5(0)	10.35	3.86	0.10	5.32
16	1:10(-1)	85(1)	5(0)	10.51	2.79	0.06	5.06
17	1:20(0)	55(-1)	3(-1)	10.51	2.15	0.06	4.87

* Indicated 5 center points

Table.S4 Analysis of variance (ANOVA) for the developed second order polynomial models on pure water extraction *Forsythia suspensa* leaf.

Source	Sum of squares	df	Mean square	F-value	P-value	
Model	5	9	0.56	5.4	0.0185	*
X_1	1.22	1	1.22	11.8	0.0109	*
X_2	4.48E-03	1	4.48E-03	0.044	0.8407	
X_3	1.03	1	1.03	9.97	0.016	*
X_1X_2	0.28	1	0.28	2.69	0.1447	
X_1X_3	0.23	1	0.23	2.25	0.1769	
X_2X_3	0.37	1	0.37	3.61	0.0993	
X_1^2	0.28	1	0.28	2.76	0.1405	
X_2^2	7.25E-03	1	7.25E-03	0.07	0.7985	
X_3^2	1.52	1	1.52	14.71	0.0064	**
Residual	0.72	7	0.1			

Lack of Fit	0.59	3	0.2	5.76	0.0619
Pure Error	0.14	4	0.034		
Cor Total	5.72	16			

Note: * represents significant difference in results ($p < 0.05$), ** represents extremely significant difference in results ($p < 0.01$).
